# Impact of Intercropped Legume Flours on the Nutritional and Textural Attributes of Wheat Cakes: A Sustainable Approach to Enhanced Nutrition

**DOI:** 10.3390/foods15152713

**Published:** 2026-08-01

**Authors:** Mehraj Fatema Mulla, Mathilde Manifacier, Sheila Alves, Karen Hussey, Antonio Martinez-Abad, Maria Castanedo, Nooshin Vahedi Kia, Ewen Mullins, Richard Lynch, Eimear Gallagher

**Affiliations:** 1 Food Quality and Sensory Science Department, Teagasc Food Research Centre, D15 DY05 Dublin, Ireland; 2 Crop Science Department, Teagasc Oak Park Crops Research Centre, R93 XE12 Carlow, Ireland; 3 Food Industry Development Department, Teagasc Food Research Centre, D15 DY05 Dublin, Ireland; 4 Food Safety and Preservation Department, Institute of Agrochemistry and Food Technology (IATA-CSIC), Avda. Agustín Escardino 7, Paterna, 46980 Valencia, Spain; 5 Food Chemistry and Technology Department, Teagasc Food Research Centre, P61 C996 Cork, Ireland

**Keywords:** intercropped, faba bean, pea, baking, germination, antinutritional factors, RFO, cake

## Abstract

The plant-based diets market has grown in popularity over recent decades and is projected to reach USD 27 billion by 2030. Innovative systems such as intercropping, defined as growing two or more crop species in proximity, are promising contributors to the advancement of sustainable agriculture by improving resource efficiency, enhancing crop resilience, and cutting down the need for chemical inputs. However, legume seed lots grown under intercropping versus monocropping systems differ in composition, which, in turn, can influence seed flour quality. Furthermore, antinutritional properties of legumes, such as phytic acid, condensed tannins (CTs), and raffinose family oligosaccharides (RFOs), limit their utilisation in product formulation. Therefore, the purpose of the study was to reduce these antinutritional compounds for utilisation of the intercropped legume flour in bakery products. A harvest of intercropped peas and faba bean mix (IM) obtained from Irish farmers was soaked (S) for 8 and 16 h and germinated (G) for 24, 48, and 72 h. Non-germinated intercropped mix (IM) was used as a control. All flours were subsequently used to substitute for wheat flour and fortify wheat-based cakes. The 72 h germinated intercropped mix (pea bean; 95.5:4.5) flour showed significantly (*p* < 0.05) lower levels of antinutritional compounds than the non-germinated intercropped flour. Raffinose, stachyose, and verbascose contents were reduced by 43%, 38%, and 46%, respectively, while phytic acid and condensed tannins decreased by 32% and 57%, respectively. The germination process also reduced the green hue (a*) from −8.37 to −6.49 and enhanced levels of soluble dietary fibre by 2.67% of the flour, while improving their suitability for bakery applications. Wheat-based cakes fortified with germinated and non-germinated intercropped legume flours showed a significant enhancement in protein and soluble dietary fibre contents. Compared with the control cake, protein content increased from 9.52 to 12.80%, while soluble dietary fibre content increased from 0.47 to 1.99% in the fortified cakes. Cakes containing up to 40% germinated flour (G72) showed comparable specific volume values 1.84–1.86 mL/g) and slice brightness (112.50–94.23) to the control cake. Additionally, cakes formulated with 40% intercropped flour showed significantly lower condensed tannin and phytic acid contents (*p* < 0.05), with reductions of 38% and 31.38%, respectively. Samples containing intercropped legume flours proved suitable for bakery applications, supporting up to 40% substitution in wheat-based cakes, and the germination process was effective in reducing antinutritional properties in intercropped legume flour fortified cakes.

## 1. Introduction

The plant-based diets market is growing and projected to reach 27$ billion by 2030 [1]. Beyond economic value, plant-based protein crops, especially pulse crops (legumes with dry edible seeds), offer numerous benefits by supplementing crop biodiversity as well as acting as a pest control and being climate-resilient. An approach known as intercropping or using multi-species systems helps to maintain a balanced agricultural environment by transforming the field into a more resilient ecosystem. Beyond simply protecting the surface from erosion, the diverse root architectures of mixed crops physically reinforce the soil structure and boost organic matter. This is particularly effective when legumes are involved; their ability to sequester atmospheric nitrogen naturally enriches the earth, cutting down on the chemical runoff often caused by synthetic alternatives [2]. Furthermore, the denser canopy cover acts as a buffer against climate volatility, preserving moisture during dry spells and preventing topsoil loss during heavy storms, thereby enhancing yield and reducing the incidence of diseases, pests, and weeds [2,3]. Farmers in Ireland are starting to see the value of mixing different crops, like combining legumes and cereals, instead of adhering to monocropping. By making these changes, Irish agriculture is moving towards a more sustainable and environmentally friendly future (Green Restoration Ireland, https://greenrestorationireland.coop/intercroppinginireland, accessed on 4 June 2025) [4].

Field peas (*Pisum sativum* L.) are the third most economically important grain legume after soybean and beans, and they have long been recognised as a valuable source of protein (with an average of 22.3%), starch, fibres, minerals, and vitamins. However, lodging risks can occur because of severe rain and wind events, which have become increasingly frequent due to climate change, directly impacting yield. Growing faba bean in the same field significantly increases the standing ability of peas, as well as their yield [5], encouraging farmer adoption and opening a new value proposition for the tillage industry. The enrichment of cereal products with legume flours is acknowledged to be an effective way to enhance their nutritional quality, primarily due to their higher protein content, improved amino acid balance, and increased levels of dietary fibre, vitamins, and minerals. In addition, legume incorporation can contribute to lowering the glycaemic response and improving overall functional and health-promoting properties of the final products [6,7,8,9,10].

However, some compounds, known as antinutrients (e.g., phytic acid, tannins, phenols, trypsin inhibitors), are naturally present in grains and legumes and are known to impair protein bioavailability and mineral absorption [11]. Phytic acid is an important antinutritional compound in legumes because of its ability to bind essential minerals. The six phosphate groups present in phytic acid have a high negative charge, allowing it to interact with minerals such as iron, zinc, calcium, magnesium, and manganese. These interactions form insoluble phytate–mineral complexes, which reduce mineral availability and limit absorption in the intestine. Therefore, high levels of phytic acid can negatively affect the bioavailability of minerals, particularly iron and zinc. However, processes such as germination can activate phytase enzymes, promoting phytate degradation and improving mineral availability [12,13]. Similarly, condensed tannins (CTs), also known as proanthocyanidins, are important antinutritional compounds in legumes because of their interactions with proteins and digestive enzymes. They can bind to proteins through non-covalent interactions, including hydrogen bonding and hydrophobic interactions, resulting in the formation of tannin–protein complexes. These complexes reduce protein availability and limit enzymatic digestion, which can decrease protein digestibility and nutrient utilisation. CTs may also inhibit digestive enzymes, further contributing to their antinutritional effects [14]. Raffinose family oligosaccharides (RFOs), including raffinose, stachyose, and verbascose, are considered antinutritional compounds in legumes because humans and monogastric animals lack sufficient α-galactosidase activity to hydrolyse their α-(1 → 6)-galactosyl bonds. As a result, RFOs escape digestion in the upper gastrointestinal tract and are fermented by colonic microbiota, producing gases that may cause flatulence, abdominal discomfort, and reduced consumer acceptance of legume-based foods [15,16]. Therefore, current approaches aim to reduce RFO levels to an optimal range rather than eliminate them completely, using strategies such as breeding, soaking, germination, malting, fermentation, and other processing methods [17].

Appropriate processing techniques may effectively decrease antinutrient levels while maintaining the functionality and integrity of the flour [18,19,20,21]. However, overprocessing (e.g., intense heat, fine milling, excessive extrusion, pressure) can denature the proteins in legumes, thereby altering overall solubility characteristics [11,20], which influences the resulting product characteristics, for example, hardness [22,23]. Overprocessing of legumes can also increase the glycaemic response when consumed [24]. In addition to nutritional considerations, it is necessary that products incorporated with legume flour exhibit good textural attributes and sensory profile to ensure consumer acceptance. This includes parameters such as colour, texture, visual appearance and shape [22,25,26]. Previous studies have shown that natural pre-treatments such as soaking reduce the level of antinutritional compounds through leaching and hydrolysis [27]. Additionally, germination for 24–48 h can increase the availability of beneficial nutrients, and the process also helps reduce antinutritional factors, thereby improving in vitro protein digestibility [19,28,29]. Although germination has been widely studied as a strategy to improve the nutritional quality of individual legumes, limited information is available on the potential of germinated flours obtained from intercropped legume (pea and faba bean seeds) systems. Furthermore, the combined effect of intercropping and germination on antinutritional compound reduction, flour functionality, and suitability for wheat flour replacement remains largely unexplored. Therefore, the objective of the present study was to evaluate flours produced from intercropped pea and faba bean seeds and to investigate the effects of soaking and germination as minimal processing approaches for improving their nutritional and functional properties. Specifically, the study first evaluated the effects of soaking and germination on the antinutritional compounds, proximate composition, and techno-functional properties of intercropped pea and faba bean flours. Subsequently, the suitability of the processed flours as partial wheat flour substitutes was assessed through their incorporation into cake formulations, with evaluation of the nutritional quality, physical characteristics, and textural properties of the resulting cakes.

## 2. Materials and Methods

### 2.1. Raw Materials

The mixed crop used was cultivated as part of a larger agronomic study over 3 seasons, aimed to optimise legume x legume intercropping, which included variety, total seed rate and species ratio as treatments. Field pea (variety ‘*Carrington*’) and faba bean (variety ‘*Louhi*’) seeds were mixed and sown in the spring, on five different Irish farms and over two seasons (2024 and 2025). The total seed rate was 100 seeds/m^2^ and the species ratio 70:30 (pea/bean). The intercrop was combine-harvested at the end of September, dried at 20 °C and cleaned. To mimic commercial conditions of flour production, 5 kg of intercropped grain for each farm was mixed. The species ratio of the resulting mixture was 95.5% pea and 4.5% faba bean, with a thousand-grain weight of 210 g and 300 g for field pea and faba bean, respectively. Soft wheat flour (Shackletons, Co., Meath, Ireland), margarine (purchased locally), water, granulated sugar (purchased locally), fresh whole eggs (purchased locally), and sodium bicarbonate (purchased locally) were used for cake preparation.

### 2.2. Flour Preparation

Three treatments of intercropped seed (pea and faba bean mix) were used in this study: (1) raw untreated intercropped mix, (2) soaked intercropped mix (1:3 intercropped mix seeds to potable water for 8 h and 16 h at room temperature; 22 ± 2 °C), and (3) germinated intercropped mix (soaking 1:3 intercropped mix seeds to potable water for 16 h at room temperature; 22 ± 2 °C and germinating at 95% relative humidity for 24 h, 48 h and 72 h). All were processed as flour. The soaked and germinated intercropped seeds were dried for 40 h at 40 °C using a Harter AIRGENEX^®^ chamber drying system (HARTER GmbH, Stiefenhofen, Germany). Raw, soaked and germinated intercropped seeds were dehulled using a sheller (Streckel & Schrader GmbH & Co. KG, Hamburg, Germany). These dehulled seeds were aspirated using a lab aspirator (Streckel & Schrader GmbH & Co. KG, Hamburg, Germany).

**Figure 1:**
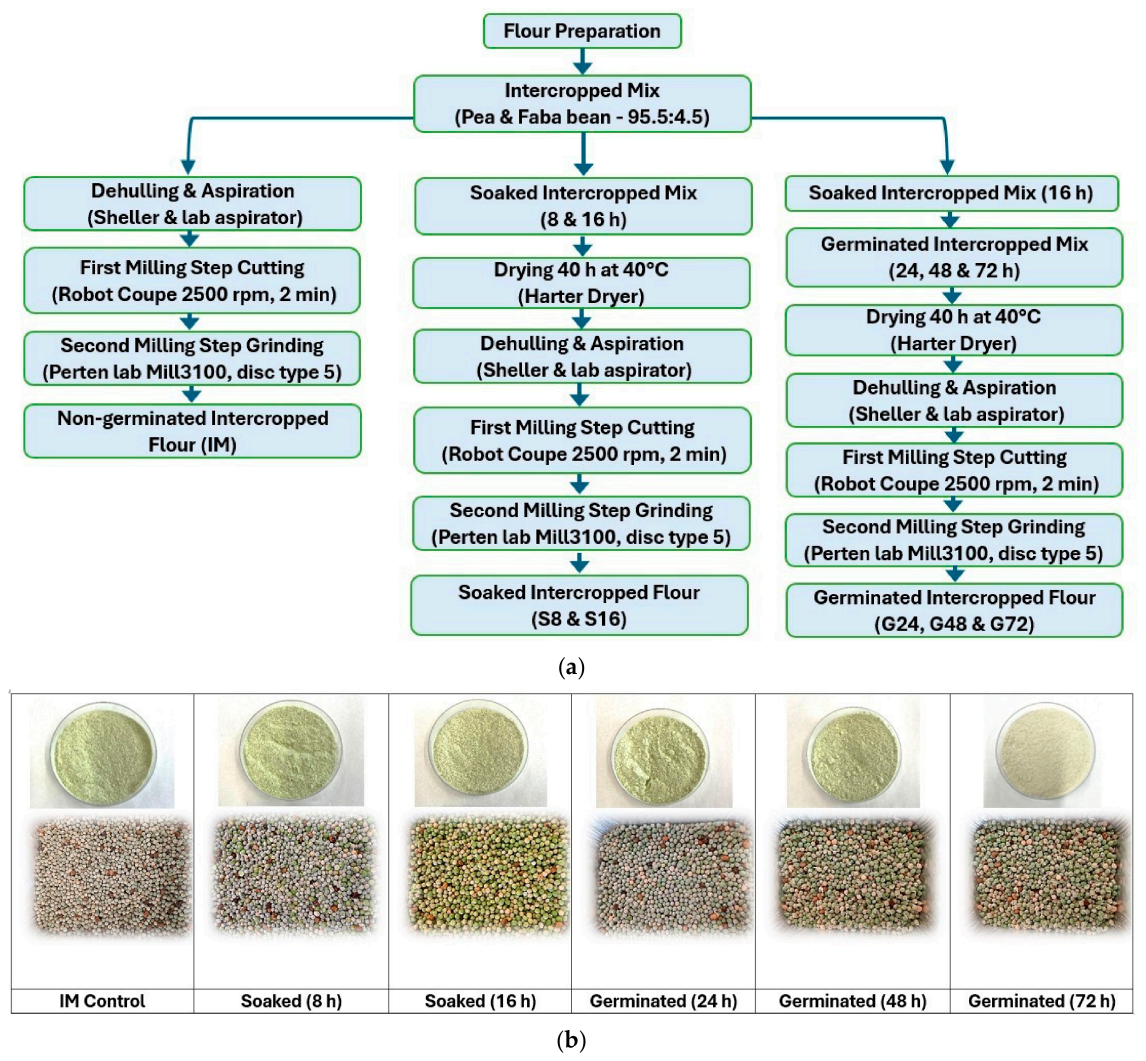
(**a**) Flowchart illustrating the preparation of flours from an intercropped pea–faba bean mixture to produce control, soaked, and germinated flours. (**b**) Representative images of the control, soaked, and germinated seeds and their corresponding flours.

Two-step milling: Dehulled and aspirated intercropped seeds (500 g) were fractured into smaller pieces using a cutter (Robot Coupe, Montceau-les-Mines, France) at 2500 rpm for 2 min. The coarse seeds were milled using a Perten Lab mill 3100 (Perten, Macquarie Park, Australia), equipped with grinding disc type 5. The milled flour was finally passed through an 850 mm sieve (Figure 1a,b).

### 2.3. Physicochemical Analysis of Flours and Cake Samples

The total proximate composition of wheat flour, intercropped mix flours, and formulated cakes was assessed. The moisture content of flour was measured following method 44–15.02 (AACC, 2000) using a Brabender oven (Mollelec Ltd., Dorset, UK) at 130 °C for 60 min [30]. For moisture determination of the cakes, a two-step method was applied, as described earlier [31]. Water activity of cake crumb slices was determined by using an Aqua Lab Lite water activity meter (Decagon Devices, Washington, DC, USA). Protein content was determined using the combustion method based on the Dumas principle with the LECO equipment (FP-628, LECO Corp., St. Joseph, MI, USA), employing a nitrogen conversion factor (Nx5.7) as per method 968.06 (AOAC, 2000) [32]. Fat content was analysed using the ORACLE Rapid NMR Fat Analyser (CEM Corp., Matthews, NC, USA) in accordance with AOAC (2005) [33]. Ash content was determined with a Gallenkamp Muffle Furnace using pre-drying at 550 °C as outlined in method 08–01.01 (Gallenkamp, Cambridge, UK; AACC) [30]. The results of protein, fat, and ash were corrected for moisture content and reported on a g/100 g dry matter (DM) basis. Soluble (SDF) and insoluble (IDF) dietary fibre was determined according to AOAC Method 991.43, using the ANKOM dietary fibre analyser (ANKOM technology, New York, NY, USA) [32]. Colour measurements of the flours were conducted using a Chroma Meter CR-410 (Konica Minolta, Basildon, UK), as described in Section 2.4.3.

The microstructure of the flour samples was examined using scanning electron microscopy (SEM). Flour samples were mounted on aluminium stubs using double-sided conductive carbon adhesive tape and sputter-coated with a thin layer of gold to improve electrical conductivity. The coated samples were observed using a SUPRA 40VP Scanning Electron Microscope (Carl Zeiss, Oberkochen, Germany) equipped with a secondary electron detector. Images were acquired at an accelerating voltage of 2 kV under high-vacuum conditions with a working distance of approximately 8–10 mm. Representative micrographs were captured at magnifications of 500× and 1000× to evaluate the surface morphology and particle characteristics of the flour samples.

#### 2.3.1. Starch Pasting Properties and RVA Gel Texture Profile Analysis

The starch pasting behaviour of germinated and non-germinated intercropped flours (pea:faba bean; 4.5:95.5) was analysed using a Rapid Visco Analyser (RVA-4, Newport Scientific Pty. Ltd., Warriewood, Australia) following Standard Profile 1, in accordance with AACC method 76–21.02 [34]. Parameters evaluated included peak viscosity, breakdown, final viscosity, setback viscosity, and pasting temperature. After analysis, the resulting pastes were sealed with a film to minimise moisture loss and stored at room temperature for 24 h to allow gel formation. The textural properties of these gels were then measured using a texture analyser (TA-XT2i, Stable Microsystems, Surrey, UK). Texture Profile Analysis (TPA) was conducted with a 20 mm cylindrical probe at 40% deformation and a test speed of 1 mm/s [35].

#### 2.3.2. Antinutritional Compounds

Phytic acid levels in the flours and cakes were measured using a Phytic Acid Assay Kit (Megazyme, Wicklow, Ireland) and a colourimetric method [36]. To extract the phytic acid, 0.5 g of sample was mixed with 10.0 mL of 0.66 M HCl and left to stir overnight at room temperature. The resulting extracts were processed according to the kit instructions. Absorbance readings were taken at 655 nm with a Cary 50 Scan spectrophotometer (Varian Inc., Palo Alto, California, USA), and results were reported as grams of phytic acid per 100 g of dry weight (g/100 g dw).

Condensed tannin content of flours and cakes was determined using the method , as described by Nagessa [37], with catechin employed as the tannin standard. A stock solution was prepared by dissolving 30 mg of catechin in 25 mL of 1% HCl in methanol. From this, a series of standard solutions of 0, 0.2, 0.4, 0.6, 0.8 and 1.0 mg/mL catechin concentration was made. Approximately 1.0 g of flour samples was weighed into 15 mL test tubes and extracted in 5 mL of 1% HCl in methanol using a mechanical shaker (MaxQ 4450, Thermo Scientific, Cincinnati, OH, USA). The extract was centrifuged at 1000 rpm for 5 min using a centrifuge (SIGMA 2-16KHL, Osterode, Germany). To develop the colour reaction, 1 mL of the supernatant was mixed with 5 mL of vanillin-HCl reagent (2.5 mL 8% HCl in methanol and 2.5 mL vanillin in methanol). The calibration of the spectrophotometer (Genesys 150, UV-Vis Spectrophotometer, Madison, WI, USA) was done with distilled water. The absorbance of all solutions was measured at 500 nm. The calibration curve was generated by plotting absorbance against concentration. To calculate the condensed tannin content, the following equation was used:

(1) $$Tannin\;\left(mg/g\right)=\frac{Sample\;absorbance-Intercept}{Slope\times Density\times Weight\;of\;sample}$$

The alpha-oligosaccharides content of the flour and developed cakes was determined using anion exchange chromatography. Briefly, 200 mg of the finely ground (<0.2 mm) sample was suspended in 1 mL deionised water and kept for 30 min at 50 °C under continuous shaking to extract alpha-oligosaccharides such as raffinose family oligosaccharides. After centrifugation at 20,000× *g* for 10 min, the supernatant was transferred to another tube, and HPLC-grade acetonitrile (ACN; Panreac Applichem, Barcelona, Spain) was added to reach 60% ACN in water. The samples were then kept at refrigeration temperature for 20 min under continuous shaking and centrifuged for another 10 min to remove the soluble protein. The supernatant was evaporated to dryness at around 50 °C, resuspended in 5 mL ultrapure water, filtered through 0.2 μm syringe filters and injected into an ICS-6000 (Thermo Fisher, Sunnyvale, CA, USA) anion exchange chromatography equipped with a Carbopac PA-1 (250 × 0.4 mm) column (Thermo Fisher, Sunnyvale, CA, USA). Raffinose, stachyose and verbascose (Merck, Darmstadt, Germany) were used for calibration.

### 2.4. Composite Flour Mixes

Soft wheat flour was used as the control flour. Six cake mixes were prepared to substitute wheat at levels of 30%, 40%, and 50% as shown in Table 1.

#### 2.4.1. Batter and Cake Preparation

A control cake formulation was used as per previous published research [31,38]. The formulation for cakes containing intercropped composite flour is seen in Table 1. All ingredients were mixed using a Kenwood mixer and K-beater attachment (Kenwood Limited, Havant, UK). In a mixing bowl, flour, sugar, egg, water, margarine, sodium bicarbonate and sugar were combined and mixed at a low speed (speed 1) for 30 s. The bowl was scraped, and the batter was further mixed at a higher speed (speed 2) for 2 min. A mini loaf tin (80 mm × 60 mm × 40 mm) was scaled with 90 g of cake batter and baked in a fan oven (Macpan MDBt8, Thiene, Italy) at 180 °C for 44 min. The cakes were cooled at room temperature, packed into polyethylene bags and stored until analysis took place. The experiment was undertaken over a 7-day period, where samples were tested at day 1, day 3 and day 7 post-baking.

#### 2.4.2. Fundamental Rheology

Batter rheological measurements were performed on a controlled stress rheometer (MCR 301, Anton Paar GmbH, Graz, Austria) fitted with 25 mm serrated probe/base parallel plates. The method used was described by [36] with slight modifications. The batter was loaded onto the rheometer after 45 min of resting at 30 °C. The excess batter was trimmed, and the edges were covered with petroleum jelly. A frequency sweep was performed with a frequency ranging from 0.01 to 10 Hz, constant shear strain (0.01%), 1 mm gap and 5 min waiting time. The plates were covered with a Peltier hood to maintain a constant temperature of 25 °C. The linear viscoelastic region was assessed initially with an amplitude sweep (ranging from 0.001 to 0.05%). The storage modulus (G′), loss modulus (G″) and tan δ (G′/G″) of the batter were recorded.

#### 2.4.3. Specific Volume and Colour

For each treatment, cakes were randomly selected and evaluated at three hours post-baking. Volume measurements and specific volume calculations were performed using a VolScan Profiler (VSP600C, Stable Micro System, Surrey, UK), with the cakes placed in an upright position during assessment.

Colour measurements of the cake crust and crumb were conducted using a Chroma Meter CR-410 (Konica Minolta, Basildon, UK), which was calibrated with a standard white reference tile. The colour parameters recorded included L* (ranging from 0 = black to 100 = white), a* (with negative values indicating green and positive values indicating red undertone), and b* (with negative values indicating blue and positive values indicating yellow hue). The Delta E (ΔE) value for crumb and crust is evaluated by using the following equation.

(2) $$∆E={\left[{\left(∆L^{*}\right)}^{2}+{\left(∆a^{*}\right)}^{2}+{\left(∆b^{*}\right)}^{2}\right]}^{0.5}$$

In this study, ΔE represented the absolute colour difference between the control wheat cake and cakes fortified with intercropped flour. Ten measurements were taken from the crust and ten from the crumb of each cake.

#### 2.4.4. Crumb Internal Texture and Structure

For crumb texture and structure analysis, each cake was cut vertically into two halves, from which 1 cm thick slices were cut. Textural attributes of the cake crumb, including hardness, chewiness, and gumminess, were evaluated using Texture Profile Analysis (TPA). From each cake, two vertical slices, each 1 cm thick, were taken from the centre region. Measurements were carried out using a TAXT2i Texture Analyser (Stable Microsystems, Surrey, UK), applying a compression test with a deformation level of 40% relative to the slice height, at a crosshead speed of 1 mm/s. A 20 mm diameter Perspex cylindrical probe (P/20P) was used for Texture Profile Analysis. A trigger force of 0.049 N was used to initiate the test. Analysis was carried out at day 1, 3 and 7 post-baking.

Structural analysis was conducted using a C-Cell digital imaging system (Calibre Instruments Ltd., Warrington, UK) equipped with dedicated analytical software. Slice evaluation included parameters such as slice brightness, dimensions (area, height, and width), cell morphology (cell count, number of holes, mean cell diameter, and wall thickness), as well as distribution features, including the presence of coarse or fine cell clusters and cell elongation patterns.

### 2.5. Statistical Analysis

All trials were replicated three times, tests were replicated three times, and mean values and standard deviations were calculated. To determine significant differences between means, the Fisher Least Significant Difference test (LSD) was performed at a 5% probability level (*p* < 0.05). All the tabulated data were statistically analysed using XLSTAT 2023.3.1 (Addinsoft, Paris, France) and the figures were generated using GraphPad Prism version 11.0.2 (GraphPad Software, San Diego, CA, USA).

## 3. Results and Discussion

### 3.1. Effect of Soaking and Germination on Antinutritional Compounds of Intercropped Flours

Phytic acid (inositol dihydrogen phosphate (IP6) or inositol polyphosphate) serves as the primary storage form of phosphorus in many seeds and grains, including peas and faba beans. It is negatively charged and exists as a globoid in protein vacuoles where it strongly binds to divalent metallic cations such as iron, zinc, calcium, magnesium and manganese to form insoluble and indigestible complexes and reduces their bioavailability [39,40]. Furthermore, phytic acid interacts strongly with dietary proteins through electrostatic attractions and hydrogen bonding, specifically targeting cationic amino acids such as lysine, histidine, and arginine. These interactions form insoluble binary complexes at the low pH of the stomach and ternary protein–mineral–phytate complexes at the higher pH of the small intestine. Because these structures are highly resistant to proteolytic enzymes such as pepsin and trypsin, they impair enzymatic hydrolysis and significantly inhibit protein digestibility [39,41]. Therefore, it is essential to reduce phytic acid in legumes via processing such as germination and soaking [42].

**Figure 2:**
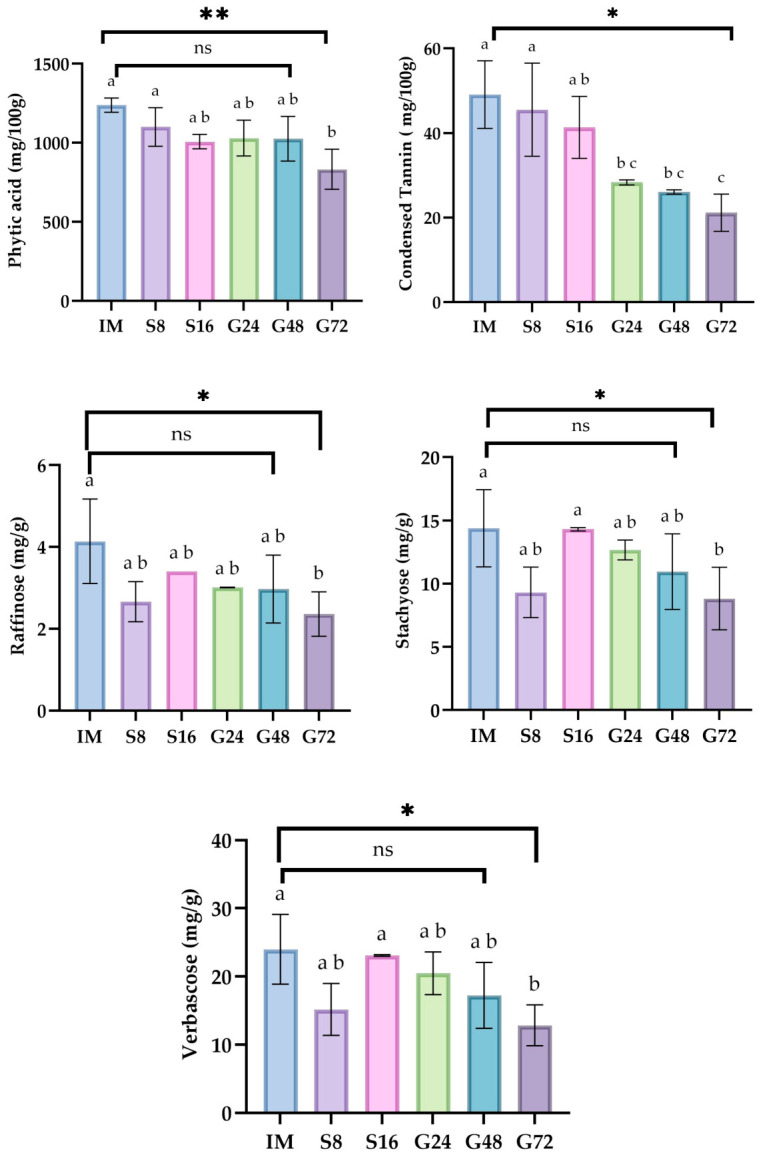
Antinutritional compounds phytic acid, condensed, raffinose, stachyose and verbascose content of flour prepared from control, soaked and germinated intercropped mix pea (95.5) and faba bean (4.5) seeds. (IM = non-germinated control, S8 = soaked 8 h, S16 = soaked 16 h, G24 = germinated 24 h, G48 = germinated 48 h, G72 = germinated 72 h). Statistical significance is indicated as follows: ns, *p* ≥ 0.05; * *p* < 0.05; ** *p* < 0.01. Different letters indicate significant differences across treatments.

Soaking (8 and 16 h) and germination (24, 48 and 72 h) of intercropped seeds were carried out. The seeds were then dried, dehulled and milled to obtain flour. The obtained flours were evaluated for their antinutritional content. The content of phytic acid in the non-germinated intercropped flour (IM) was comparable to earlier reports on pea and faba bean [43]. From Figure 2, it can be observed that soaking did not significantly (*p* > 0.05) reduce the phytic acid content. However, a reduction in phytic acid content was observed in the germinated flour and the decrease was more pronounced with the increase in germination time. After 72 h of germination, the phytic acid content of intercropped flour (G72) decreased by 32% (*p* < 0.05). Similarly, Marzo et al. (1998) studied the effect of soaking and germination on three pea (*Pisum sativum* L.) cultivars, i.e., Renata, Solara and Ballet, and their effect on phytic acid content [44]. These authors stated that the different varietal characteristics such as seed coat structure, size and density of beans and their intrinsic phytase activity are important factors influencing phytic acid reduction during germination [38]. In another study, the authors attributed the reduction in phytate (phytic acid) content during germination to the action of the phytase enzyme, which degrades phytate during the germination of cereals and legumes. Further, they reported that phytate is formed during seed maturation, and differences in its content may reflect variations in seed maturity at harvest, while its reduction during germination is mainly driven by intrinsic phytase activity [45]. Therefore, the observed reduction in phytic acid during germination can be attributed to the activation of the naturally occurring enzyme phytase during the germination process, which breaks down phytic acid to release phosphorus and other complexed minerals, and these nutrients fuel the rapid growth of the seedling [13,46].

Condensed tannins (CTs) are antinutritional polyphenolic compounds that can affect nutrient digestibility and bioavailability through their strong interactions with proteins, carbohydrates, and minerals. In published studies involving animals, the presence of CTs diminished growth rate, the bioavailability of mineral elements, and decreased feed efficiency in rats, swine, poultry, and ruminants [47]. The recent literature highlights that these compounds exhibit a much greater tendency to bind with proteins than other food polymers due to the high hydrogen bond affinity of the carboxyl oxygen of the peptide group. The formation of these complexes is highly dependent on environmental variables such as pH, temperature, and concentration, and once established, they effectively sequester dietary proteins and inactivate digestive enzymes and microbial cells. By disrupting microbial fermentation and slowing the digestion of ruminal protein and dry matter, these interactions are directly responsible for growth depression, low protein digestibility, decreased amino acid availability, and increased faecal nitrogen excretion [48,49].

Condensed tannins (CTs) content of the non-germinated (IM) flour was 49.07 ± 8.9 mg/100 g. The CT content previously reported for green pea varieties has been in the range of 23 mg/100 g to 61 mg/100 g and for faba bean, ranges from 95.8 mg/100 g to 195 mg/100 g [50,51,52,53]. Our readings are in the middle, not too low. This could be due to the dehulling process, as tannins and phenolics are mainly in the hull/testa of faba bean; therefore, removing the hull substantially reduced their levels [54]. There was a significant decrease in the CT content of G72 due to the germination process, with reductions of approximately 42, 46 and 59% observed after 24, 48, and 72 h of germination. Other studies have also found a 79% reduction in CTs in faba bean following 3 days of germination. The author stated that the reduction in tannin levels during germination is primarily due to the increasing activity of poly phenol oxidase (PPO) and other catabolic enzymes that hydrolyse and transform tannins into other products for example PPO oxidises phenolic tannins (mainly o-diphenols) to highly reactive quinones and these quinones then polymerise or bind to proteins and other macromolecules, so they no longer appear as extractable “tannins” in analysis [39,55]. Similarly, previous studies have reported reductions in antinutritional factors during the germination of legumes such as chickpea, faba bean, cowpea, and soybean. This reduction has been attributed to the activation of enzymes, including polyphenol oxidase and other catabolic enzymes, which oxidise and hydrolyse tannins, thereby lowering their concentration in the seeds [47]. Additionally, this decline was also attributed to the physical leaching of these compounds into the soaking water [39].

Plants synthesise a wide range of primary and secondary metabolites that play critical roles in growth, development, and stress adaptation. Among these, the raffinose family oligosaccharides (RFOs), composed of α-galactosyl derivatives of sucrose such as raffinose, stachyose, and verbascose, function as reserve carbohydrates and contribute to seed germination, desiccation tolerance, and abiotic stress resistance [16]. These are soluble carbohydrates widely found in plants, particularly in legume seeds such as soybeans, lentils, and beans. However, elevated concentrations of RFOs in legumes are considered nutritionally undesirable, as the intestinal mucosa of humans and monogastric animals lacks the specific hydrolysing enzyme α-galactosidase (AGAL). Therefore, these oligosaccharides cannot be digested in the upper gastrointestinal tract. Recent findings indicate that RFOs pass into the lower intestinal tract, where colon microflora metabolise them via anaerobic fermentation. This process produces excess carbon dioxide, hydrogen, methane, and traces of short-chain fatty acids (SCFAs), leading to flatus accumulation and discomfort. In addition, the presence of undigested RFOs may contribute to osmotic imbalance within the intestine, interfere with the absorption of water and nutrients, and accelerate digesta transit rate, thereby reducing nutrient utilisation efficiency and true metabolizable energy (TME) [15].

From Figure 2, it can be observed that the RFOs (raffinose, stachyose, verbascose) content decreased following germination for all flours. The contents of raffinose, stachyose, and verbascose were significantly reduced (*p* > 0.05) after 72 h of germination in the intercropped flour samples, with decreases of 43%, 38%, and 46%, respectively. Previous studies on faba bean and peas also reported a considerable reduction in RFO levels with germination [56]. For example, Wei et al. (2022) observed a ∼10% decrease in raffinose content of faba bean cultivars after 12 h of soaking and 100% elimination of raffinose due to germination at 24 h, 48 h and 72 h [57]. However, soaking did not show a reduction in stachyose level, while a significant (*p* > 0.05) decrease was observed after 24 h of germination. The author stated that seed germination induces alpha-galactosidase activity, which could promote the enzymatic hydrolysis of RFOs [57]. Another study on peas stated that the breakdown of RFOs (started during seed imbibition) continued in the cotyledons up to the 7th day of seed germination [58]. The reduction in RFOs in the intercropped flour samples during germination is likely due to their breakdown by α-galactosidase (α-GAL) enzymes, which hydrolyse RFOs into sucrose and galactose. These sugars are then used as energy and protective compounds during the transition from seed to seedling, leading to a sharp decrease in RFO content in germinated flours [15].

However, the reduction in RFO in the intercropped flour did not occur at a similar rate. The extent of this decrease depends on the initial distribution of RFOs (cotyledon vs. axis) and seed structure, which affect how quickly RFOs are mobilised; differences in glycolysis and the TCA cycle in embryonic axes reflect variations in the seed’s energy demand; and species-specific rates of α-galactosidase activity that affect the level of RFO in germinated seeds collectively [15,17,59].

As 72 h germinated flours showed the lowest levels of antinutritional compounds (phytic acid, CTs and RFOs), these samples were selected alongside the non-germinated control flour for the development of composite flour blends used in wheat-based cake production.

### 3.2. Nutritional Composition and Techno-Functional Properties of Intercropped Flours

The proximate composition and functional properties of non-germinated (IM), soaked (S8 and S16) and germinated (G24, G48 and G72) intercropped flours are summarised in Table 2. The moisture content of the flours ranged from 6.23% to 11.93%, while protein content varied between 23.12% and 24.96%. No significant differences were observed in fat content among the samples, whereas ash content ranged from 2.48% to 2.70%. The highest level of protein 24.96% was observed in the intercropped flour following 72 h of germination. Earlier studies have reported a similar increase in the protein content of germinated flours, attributing this change to the respiration processes during sprouting, which consume carbohydrates and lipids while promoting the synthesis of new protein compounds and enzyme proteins in germinating legume seeds, ultimately resulting in increased amino acid production [60,61].

The germinated (G24, G48 and G72) intercropped flour showed significantly higher soluble dietary fibre (*p* < 0.05) when compared to the unprocessed and soaked intercropped flour. This is consistent with the previous results where the authors observed an increase in the SDF content of the germinated peas and chickpeas [62,63]. During germination of legumes, hydrolytic endogenous enzymes (xylanases, cellulases, hemicellulases, and pectinases) are activated that remodel cell walls and partially solubilise structural polysaccharides (arabinoxylans, cellulose, hemicellulose, pectin) into shorter, more water-soluble chains, increasing SDF and lowering IDF [62]. The increase in SDF content in the intercropped flour as a result of germination could be attributed to the distinct enzymatic activity during the subsequent germination process [61,64].

WHC of flour depends on the function of starch, protein and fibre. In Table 2, it can be observed that the WHC of non-germinated intercropped flour (IM) was significantly higher (*p* < 0.05) than that of soaked (S8 and S16) flour. The lowest WHC was observed during germination (24 h); amylases and other enzymes hydrolyse starch into smaller sugars and degrade some dietary fibre, which can reduce swelling power and the number of hydrophilic (water-binding) sites, leading to lower WHC in germinated flours [60,65]. The lower WHC of the flour influences the water retention capacity, which can increase the moisture loss during baking and result in a dense, dry cake [66].

The colour (a* and b*) of the intercropped flour was significantly (*p* < 0.05) affected by the germination process. The a* (redness/greenness) values of intercropped flour decreased from −8.37 to −7.50 after 16 h of soaking and −6.49 after 72 h of germination (*p* < 0.05). The reduction in flours’ a* green undertone is due to the activation of chlorophyllase and other chlorophyll-degrading enzymes during soaking and germination, which break down chlorophyll and, consequently, reduce green undertone [67].

**Figure 3:**
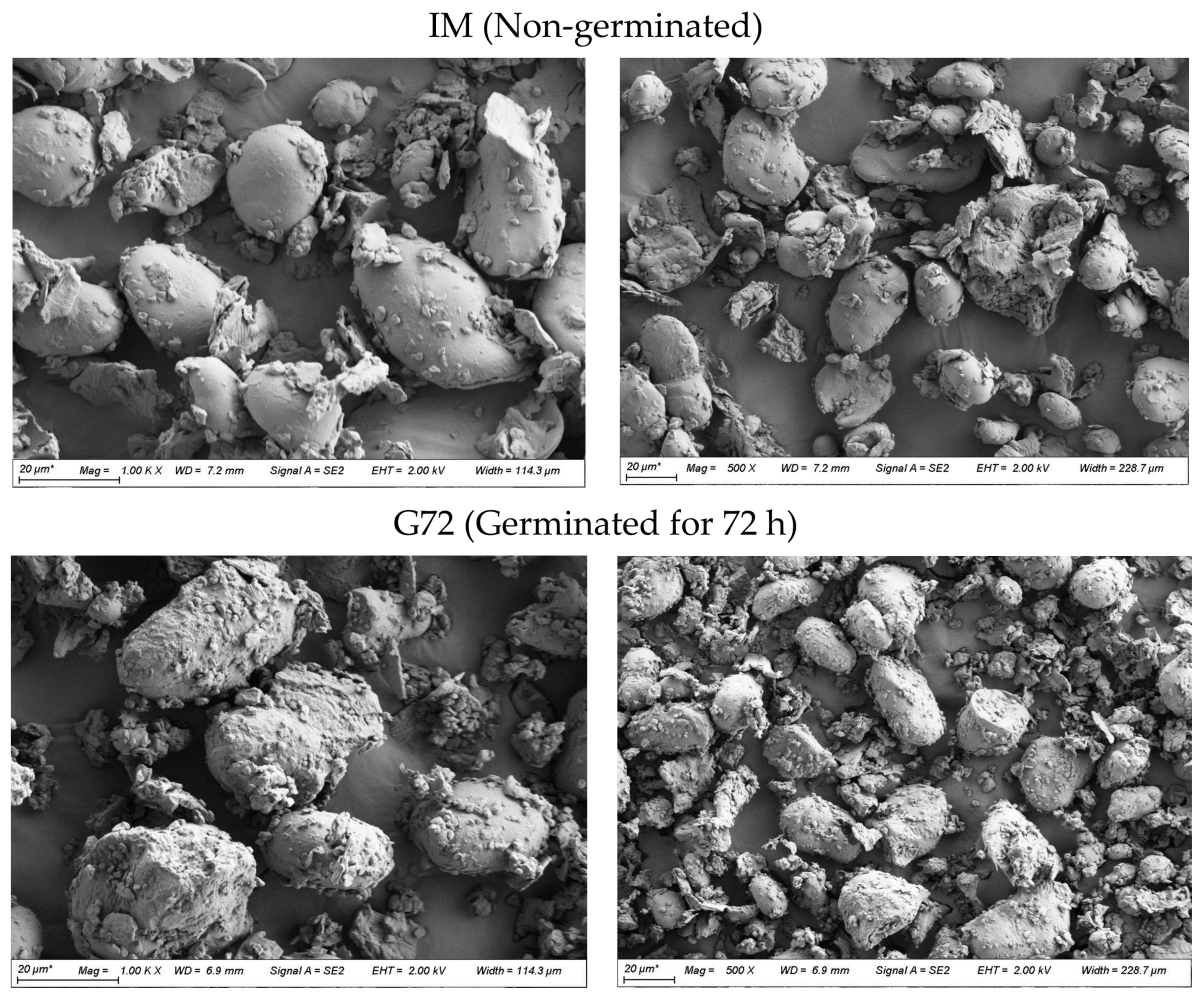
Scanning electron microscopic images of non-germinated (IM) and 72 h germinated (G72) intercropped flours. The asterisks mark the scale bars in each panel.

A Rapid Visco Analyser (RVA) is a specialised, rotational rheometer used to assess the pasting properties of flour or starch. It mimics a cooking process by subjecting a flour–water slurry to a controlled cycle of heating, holding, and cooling while continuously stirring [68]. RVA analysis illustrated a significant (*p* < 0.05) decrease in peak viscosity for intercropped flour after 72 h germination. This decrease in peak viscosity could be attributed to the structural changes in starch and protein during germination. The peak viscosity of an RVA curve depends on the integrity and swelling capacity of the starch granules of the sample; therefore, enzymatic hydrolysis, specifically by amylases, breaks down these granules and lowers the resulting viscosity. This aligns with findings by Xu et al. (2019), who observed a similar trend in yellow peas following germination and attributed it to the enzymatic degradation of native starch [69]. Further, higher levels of protein and fibre associated with the starch granule surfaces (see Figure 3) can restrict granule swelling and network formation, resulting in lower peak and final viscosities and the formation of weaker gels by the end of the RVA test [69].

Final viscosity of the starch paste reflects the ability of a flour–water system to form a gel and undergo starch retrogradation during cooling. The final viscosity of the intercropped flour pastes increased significantly (*p* < 0.05) for soaking and germination up to 48 h but decreased significantly (*p* < 0.05) after further germination to 72 h. These findings align with previous studies demonstrating that a six-day germination period for yellow peas significantly altered pasting characteristics [69]. Specifically, the non-germinated (day 0) yellow pea flour exhibited the highest final viscosity, with a consistent decline in viscosity as the germination progressed. This indicates that with longer germination, the viscosity of the paste can drop, as starch is degraded due to the increased endogenous alpha-amylase activity which occurs during germination [65,69]. Peak time on the RVA curve represents the duration required for a flour–water slurry to reach its maximum (peak) viscosity during heating. A significant increase in peak time was observed due to soaking, while the graph also showed a significant (*p* < 0.05) decrease for the germinated flour. This could be due to enzymatic modification during germination, which may cause partial hydrolysis of starch leading to a disruption in hydrogen bonds of starch molecules. These can be detached easily during heating in RVA analysis [29,70].

The textural characteristics of the RVA gel provide important insights into starch retrogradation over a 24 h period following the RVA test. Retrogradation is a primary factor contributing to cake staling or hardening; therefore, lower retrogradation rates are desirable in cake production. As shown in Table 2, the gel hardness of both soaked (8 and 26 h) and moderately germinated (24 and 48 h) intercropped flour gel was higher (*p* < 0.05) than that of the non-germinated control. This parameter is influenced by the final viscosity and the association of amylose chains. The firmer gel formation for the soaked flour could be due to leaching of soluble sugars during soaking and alignment of amylose chains more tightly [71]. However, a significant reduction in hardness was observed for intercropped flour which was germinated for 72 h. This reduction may be attributed to enzyme-mediated starch degradation and changes in the association between starch granules and surrounding protein/fibre components, as indicated by the morphological alterations observed in the SEM images (Figure 3). A possible explanation is that 24–48 h of germination represents an optimal period of partial modification, during which biochemical changes improve gel formation without causing excessive degradation of structural components. During this stage, limited hydrolysis of storage proteins and carbohydrates may enhance molecular interactions, increase water-binding capacity, and promote the development of a stronger gel network. However, after 72 h of germination, prolonged enzymatic activity, including amylolysis and proteolysis, together with structural changes associated with seedling development, may result in greater degradation of the matrix and reduced gel hardness. Therefore, early germination may improve gel-forming properties through controlled biochemical modification, whereas extended germination may weaken the gel structure due to excessive breakdown of seed storage components [72]. These structural modifications may reduce starch granule integrity and limit starch retrogradation, which could contribute to delayed staling in cake products. Consequently, germinated flour may form a softer and less rigid gel structure compared with non-germinated flour [29].

### 3.3. Microstructure of Intercropped Flours

The SEM images of the non-germinated and 72 h germinated intercropped flour can be seen in Figure 3. The integrity of the starch surface remained unchanged after 72 h of germination with no noticeable alteration in granule structure [73]. However, the germinated flour showed more protein and fibre attached to the starch granule. Similar observations were stated earlier for germinated pigeon pea flour, and the author attributed this to the protein and fibre hydrolysis during germination [29].

### 3.4. Properties of Cake Batter and Cakes Fortified with Intercropped Flours

#### 3.4.1. Cake Batter Rheology

**Figure 4:**
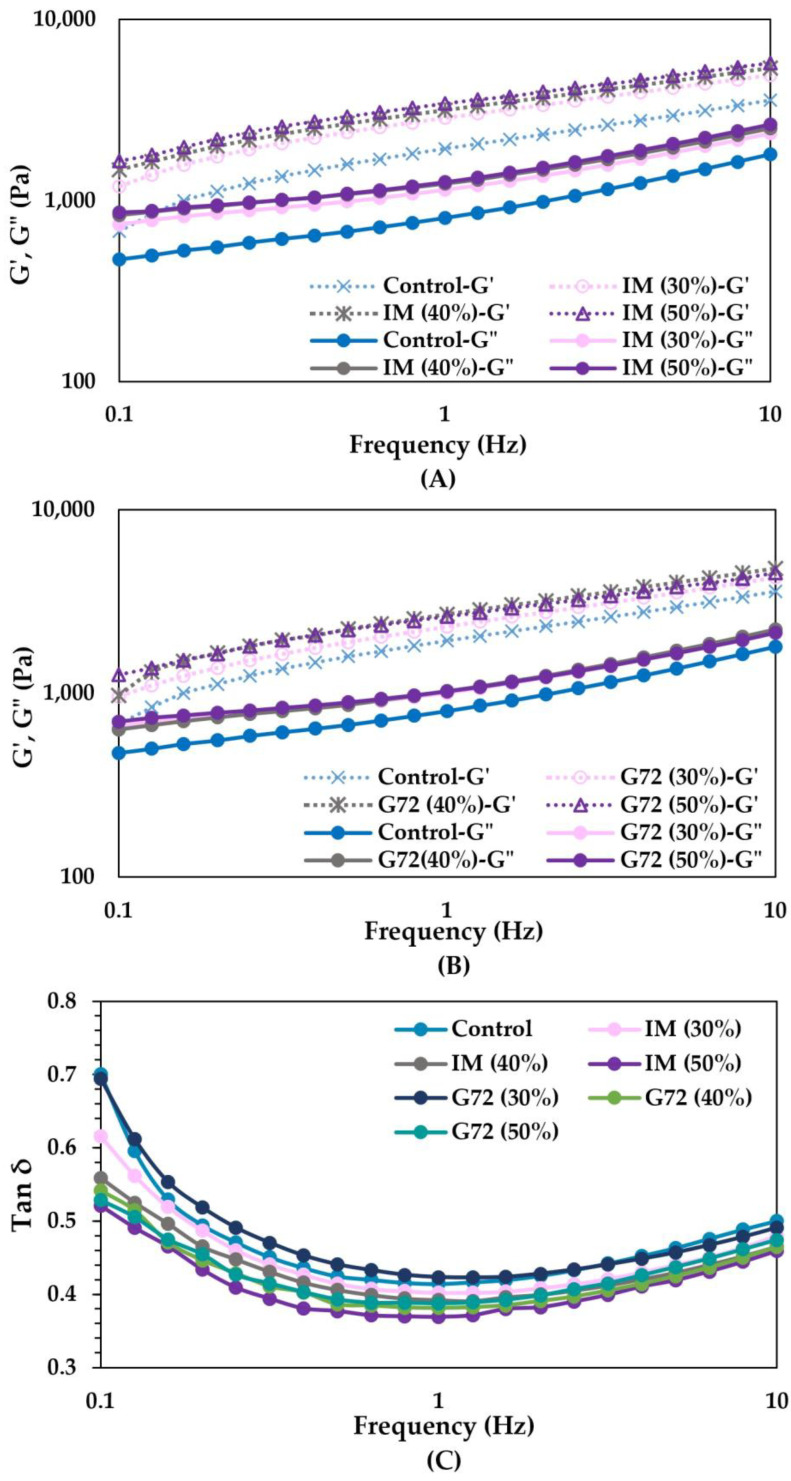
Storage modulus G′, loss modulus G″ and Tan δ of batter from wheat flour substituted with (**A**) non-germinated intercropped flour (IM); (**B**) germinated intercropped flour (G72) at 30%, 40% and 50% levels; (**C**) Tan δ values of germinated and non-germinated intercropped flour incorporated with different amounts, i.e., 30%, 40% and 50% in cake batter and its comparison with control wheat flour batter.

The mechanical rigidity of cake batter samples prepared from wheat flour (control), 72 h germinated intercropped flour (G72) and non-germinated intercropped flour (IM) (Table 1) was evaluated at 25 °C using frequency sweep tests (Figure 4A–C). The ANOVA analysis was carried out and is presented in the Supplementary Data (Figure S1). As the frequency increased from 0.1 to 10 Hz, both dynamic moduli exhibited frequency dependence, with values increasing at higher incorporation levels (30–50%). The batter samples exhibited a solid-like behaviour (G′ > G″) irrespective of the incorporation levels and flour type (IM and G72) throughout the studied frequency range, indicating that elastic properties of the batter samples were more prominent than the viscous properties [74]. The storage modulus (G′), which represents the elastic property of the batter, increased significantly (*p* < 0.05) with intercropped flour fortification up to the 40% level. However, further incorporation (i.e., 50%) significantly (*p* < 0.05) reduced the storage modulus for germinated intercropped flour fortified batter. This indicates that the batter becomes more structured and viscoelastic, which can lead to denser, harder cakes with lower volume [75]. A concurrent increase in the loss modulus (G″), an indicator of viscous behaviour, was observed in all fortified cake batters compared with the wheat control (Figure 4 and Figure S1). This increase in viscoelastic moduli is consistent with the findings of Sallam et al. (2021), who reported that cake batter fortified with green pea waste flour (GPWF) exhibited increased protein content and reduced moisture content due to the high fibre content of GPWF [76].

Tan δ or loss tangent, representing the ratio of viscous (G″) and elastic (G′) components of the batter, was lower than 1 for all the analysed samples (Figure 4C). As the level of IM and G72 substitution increased, Tan δ values significantly (*p* < 0.05) decreased. A similar reduction in Tan δ from 0.6 in the reference formula (S0) to 0.3 at the highest fortification level (S40) was reported by Bustillos et al. (2020) for sponge cake batter fortified with pea proteins. The authors noted that this trend indicates a shift toward a more elastic, solid-like nature, where the storage modulus (G′) increasingly dominates over the viscous, liquid-like properties (G″) [77]. This behaviour is essential for high-quality batter, as it enhances gas retention and structural stability during processing and baking.

#### 3.4.2. Cake Nutritional Composition

Wheat flour was replaced with 30%, 40%, and 50% levels of both non-germinated (IM) and 72 h germinated (G72) flours in a cake formulation. Statistical analysis showed no significant difference in the moisture and fat content between the control and the fortified cakes on day 1. However, the inclusion of intercropped flours positively impacted (*p* < 0.05) the protein and ash profiles (Table 3). The highest protein concentrations, of 12.43% and 12.80%, were found in cakes substituted with 50% non-germinated and germinated intercropped flour (IM and G72, respectively), representing a substantial increase when compared to 9.52% protein found in the wheat-only control. Dietary fibre content increased progressively with higher substitution levels. The cakes with 50% IM exhibited the highest total and insoluble fibre at 7.50% and 6.04%, respectively, while the 50% G72 fortified cakes peaked in soluble fibre at 1.99% as compared to the wheat-only control containing 0.98%. These results align with previous findings which showed that substituting part of the wheat flour with pea or faba bean (in various forms) can significantly raise total and insoluble fibre content in cakes and similar baked goods, often doubling or tripling fibre concentration [78].

#### 3.4.3. Cake Specific Volume, Crust and Crumb Colour

In Table 4, it can be observed that cakes prepared with a 50% substitution level of wheat flour with intercropped flour exhibited the lowest specific volume among the samples tested. In contrast, 30% incorporation of germinated and non-germinated flours yielded cakes with higher specific volumes in comparison to the control cake, with values of 2.09 and 1.93 mL/g versus 1.84 mL/g for the control cake. This improvement is likely attributed to the synergistic interaction between the flour components (starch and protein) and the gluten protein, which improves aeration and structural stability. Notably, samples containing 40% of germinated and non-germinated (G72 and IM) flours maintained specific volumes comparable to the control, suggesting that moderate fortification can effectively support volume retention. However, exceeding this threshold leads to a decline in specific volume (at 50% substitution). Similar observations were reported by Aydogdu et al. (2018) for cake batter substituted with pea fibre, who stated that dietary fibres may interfere with gluten network formation and dilute functional gluten proteins, thereby disrupting the starch–gluten matrix and reducing gas retention capacity, ultimately resulting in lower cake volume [75].

The changes in the colour values (L*, lightness), (b*, yellow undertone), and (a*, red/green undertone) of the intercropped flours (IM and G72) fortified cakes (crust and crumb) were determined, and the total colour difference delta E (ΔE), relative to the control, is presented in Table 4. The ΔE values of both crust and crumb of fortified cakes prepared with 30% non-germinated and germinated (IM and G72) flours were significantly higher than those of the control cake. However, the ΔE values increased significantly (*p* < 0.05) with higher incorporation levels of both germinated and non-germinated (IM and G72) flours. This could be attributed to the Maillard reaction, i.e., the formation of brown pigments in legume flour due to reactions between reducing sugars and amino groups during baking, which impart the final colour to the cakes [79]. However, the highest ΔE values were observed in the crust of cakes substituted with the 50–germinated (G72) flour. This is likely due to the compositional changes induced by germination. During germination, endogenous enzymes hydrolyse starch and proteins, increasing reducing sugars and free amino acids, which are key Maillard precursors [80]. At the 50% substitution level, more of these precursors are incorporated into the cake batter, and during baking they promote greater formation of Maillard brown pigments, especially in the crust where thermal exposure is highest. Similar observations have been reported in previous studies on bread, where colour values became progressively darker with the inclusion of higher levels (e.g., 20%) of germinated or fermented cowpea flour. Authors attributed this effect to enhanced Maillard reactions during baking, associated with the high lysine content of cowpea flour [81].

#### 3.4.4. Crumb Structure

Internal structural aspects of the cakes were determined by the light reflectance from the crumb calculated with image analysis software associated with the C-Cell DIA (Digital Imaging Analysis) system (Table 4). Higher brightness values typically indicate a finer crumb texture and a more uniform distribution of air cells. From Table 4, it can be observed that the control cake samples demonstrated significantly higher slice brightness values (*p* < 0.05). This could be due to a higher proportion of WF in the control, which also has a greater L* value in comparison to germinated (G72) and non-germinated (IM) intercropped flours. Additionally, the incorporation of non-germinated flours at levels of up to 40%, and germinated samples of up to 50% inclusions, resulted in cake slices with a visibly bright appearance. The cell area (%) remained comparable to the control wheat cake at substitution levels up to 40% for both (G72 and IM) germinated and non-germinated flours. Cell formation and diameter are intrinsically linked to the integrity of the gluten network. At 50% substitution, the batter stability is compromised, as evidenced by the significantly (*p* < 0.05) lower (G′) values. This structural weakness causes the small air cells to collide and merge into larger, irregular pockets or simply collapse, as the fragile matrix can no longer support the gas pressure. Consequently, this leads to a denser cake with a less uniform crumb structure compared to the control and lower substitution levels [82].

#### 3.4.5. Crumb Moisture Content and Texture During Storage

**Figure 5:**
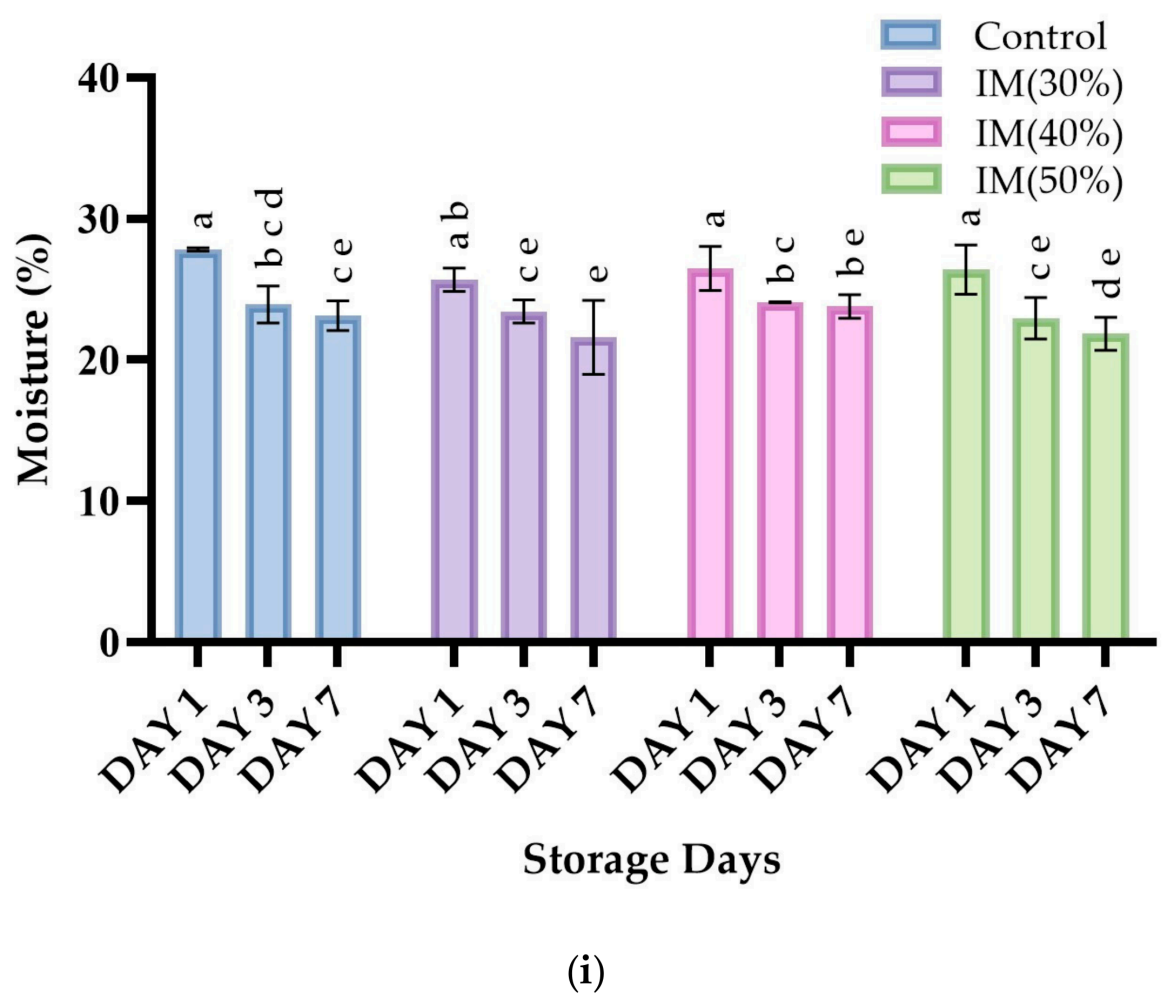

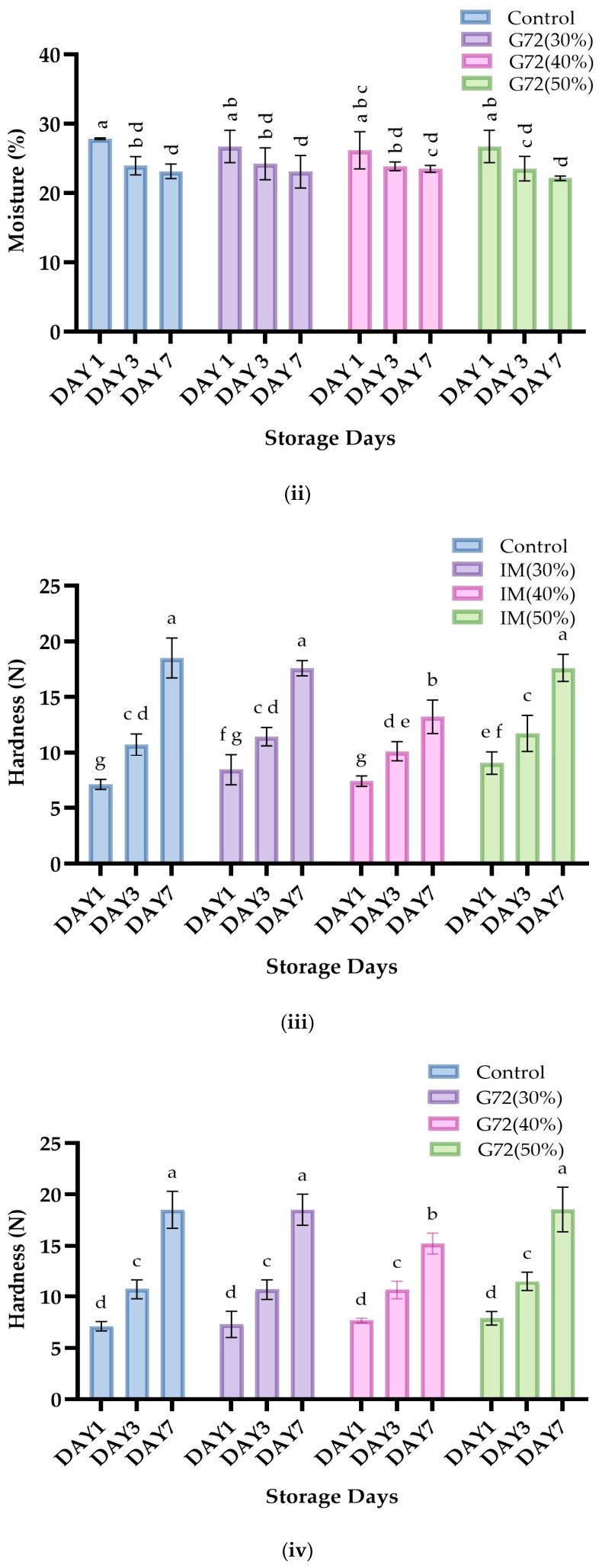
Moisture content (**i**,**ii**) and hardness (**iii**,**iv**) of cake crumb with non-germinated and germinated intercropped flours (IM and G72) at 30%, 40%, and 50% substitution, compared with control wheat sponge cake during 7 days of storage at room temperature. Different letters indicate significant differences across storage days within the same treatment.

Crumb moisture content and hardness typically share an inverse relationship, as moisture levels directly influence textural attributes, particularly the softness of the cake. The moisture content of the cakes over a 7-day storage period is illustrated in Figure 5. The changing moisture content of all cake samples remained non-significant until the third day of storage. Significant changes emerged by the seventh day, with the 50% substituted cakes (IM and G72) exhibiting the lowest moisture levels, ranging from 21.85% to 22.13%. The lowest rate of moisture loss was 0.38% for G72 at 40% incorporation level as compared to the wheat control (0.67%). This signifies that the cakes with 40% G72 retain moisture and slow down the staling process at a lower rate than control cakes.

Similar findings were observed in previous studies for cakes substituted with chickpea-based emulsions and pea flour that showed reduction in moisture loss during storage [83,84]. Conversely, the highest rate of moisture loss was observed for the 50% non-germinated IM fortification level. This accelerated decline is likely due to the 50% substitution creating a weak, porous matrix that cannot trap moisture as effectively as the 30% to 40% substituted cakes and allows water to migrate more easily toward the crust and evaporate. Similar results were found by Sanfilippo et al. (2024), who explained that breads with higher legume levels showed a more rapid moisture decline due to enhanced water migration at higher substitution levels [85].

In food texture analysis, hardness is defined as the peak force measured during the initial compression of a Texture Profile Analysis (TPA) curve (Figure 5). This parameter assesses a product’s resistance to deformation and reflects the force required for the initial bite. According to Figure 5, cakes fortified with 40% intercropped (IM and G72) flour exhibited significantly (*p* < 0.05) lower crumb hardness (day 1 and day 7) than the other formulations. This suggests that 40% substitution provided an optimal balance between wheat flour and intercropped legume flour. At this level, the fibre and protein components of the legume flour likely improved water retention while maintaining the integrity of the cake matrix, resulting in a softer crumb [86]. At 30% substitution, the amount of legume flour may not have been sufficient to achieve the same effect. In contrast, at 50% substitution, the higher level of flour replacement may have altered the starch–protein matrix, reducing its ability to maintain a soft and cohesive crumb. After 7 days of storage, cakes made with 50% non-germinated intercrop mix had a hardness of 17.62 N, whereas those with germinated intercropped flour (G72) were harder, at 18.53 N. Similar observations were reported by researchers in cakes fortified with pea fibre, who stated that higher dietary fibre concentrations (e.g., 10% or more) can increase hardness (accelerate staling) compared to lower concentrations (e.g., 5% or less) due to enhanced water binding and starch retrogradation [75].

#### 3.4.6. Cake Antinutritional Compounds

**Figure 6:**
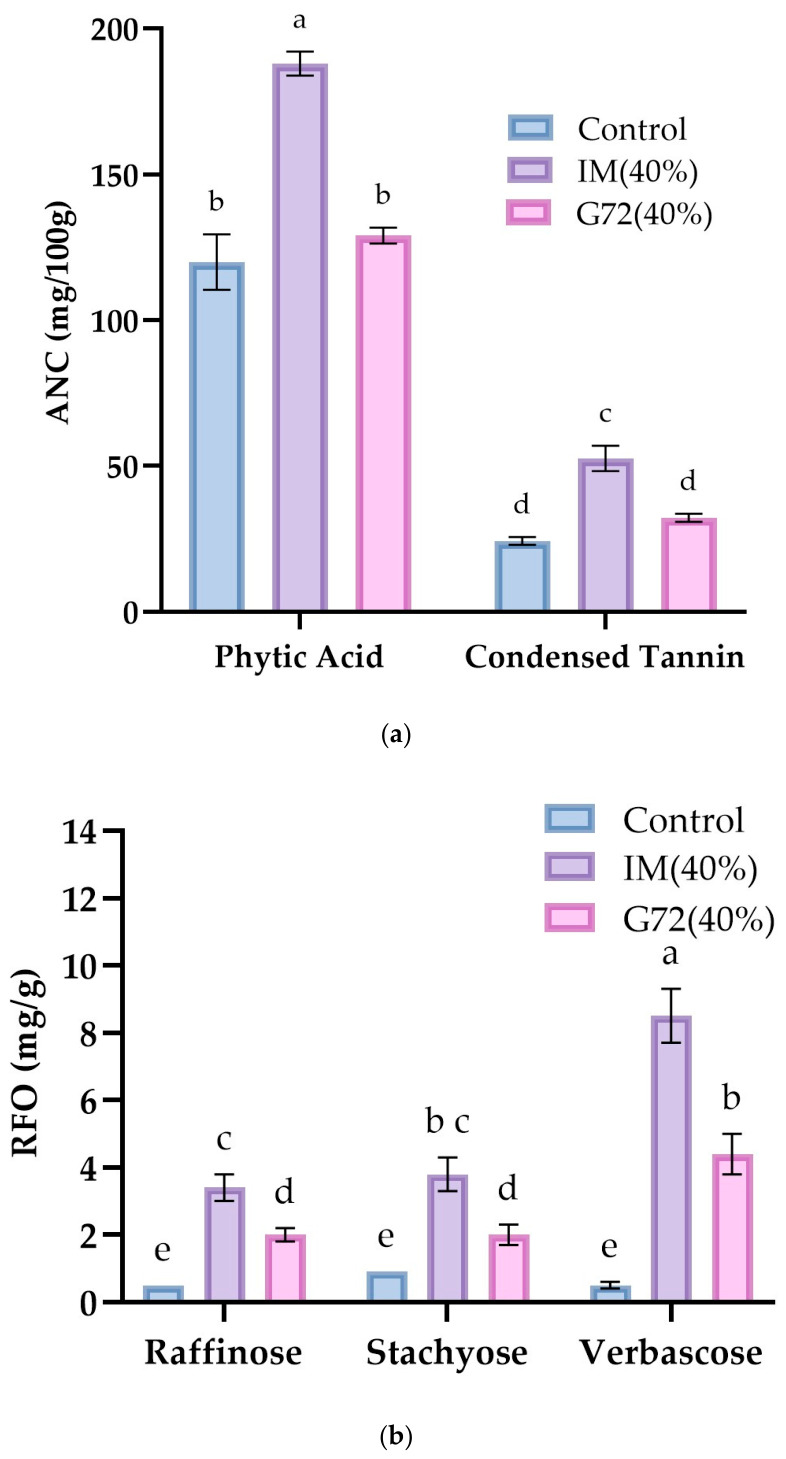
Antinutritional compounds (ANCs) phytic acid, condensed tannins and raffinose family oligosaccharide (RFO) content of cakes prepared with control wheat flour and 40% substitution of non-germinated (IM) and germinated (G72) intercropped flours. (**a**) Antinutritional compounds (Phytic acid and condensed tannin), (**b**) Raffinose family oligosaccharides (Raffinose, stachyose and verbascose). Different letters indicate significant differences between the corresponding parameters.

As the 40% substitution of IM and G72 maintained acceptable moisture levels and crumb texture throughout the storage period, these specific formulations were selected for further evaluation of antinutritional profiles. Cakes prepared with 40% non-germinated intercropped flour (IM40%) had significantly (*p* < 0.05) higher levels of antinutritional compounds, including phytic acid, condensed tannins (CTs), and raffinose family oligosaccharides (RFOs). In contrast, incorporation of 40% germinated flour (G72) resulted in significantly (*p* < 0.05) lower levels of these antinutritional compounds, which were comparable to those of the wheat-based control cakes for phytic acid and condensed tannin levels (Figure 6). The phytic acid level in control cakes (119.91 ± 9.4 mg/100 g) was similar to previously reported studies (84.09 mg/100 g to 290.82 mg/100 g) [87]. Similarly, the CT content of the control cakes (24.28 ± 0.68 mg/100 g) was consistent with the previously reported values for wheat flour-based cakes [22]. The incorporation of non-germinated intercropped flour (IM) increased the levels of phytic acid, CT, and RFOs in the cakes. In contrast, cakes prepared with 40% G72 showed reductions of 31% in phytic acid, 38% in CT, 41% in raffinose, 47% in stachyose, and 48% in verbascose compared with cakes fortified with 40% non-germinated IM. These reductions can be attributed to the lower concentrations of antinutritional compounds in the germinated flour. Furthermore, the antinutritional compound contents of cakes prepared with G72 were comparable to those of the control cakes, whereas cakes fortified with non-germinated IM contained significantly (*p* < 0.05) higher levels. These findings indicate that germination is an effective approach for reducing antinutritional compounds and improving the suitability of intercropped legume flour for bakery product fortification.

## 4. Conclusions

The present study demonstrated that a 72 h germination process is an effective minimal bioprocessing strategy for enhancing the nutritional and functional properties of intercropped pea and faba bean flours for bakery applications. Germination significantly reduced major antinutritional compounds, including phytic acid, condensed tannins, and raffinose family oligosaccharides, while improving the suitability of the flours for use as partial wheat flour substitutes. Incorporation of both germinated and non-germinated intercropped flours maintained stable cake batter rheology, with fortification levels up to 40% producing cakes with desirable specific volume, crumb structure, texture, and colour. Although higher substitution levels (50%) weakened the batter network, cakes containing 40% germinated flour achieved an optimal balance between enhanced nutritional quality and desirable technological properties. Overall, the findings demonstrate that germinated intercropped legume flour is a promising sustainable ingredient for developing protein- and fibre-enriched bakery products with reduced antinutritional content while maintaining product quality. This approach supports the increased utilisation of intercropped protein crops and contributes to the development of more sustainable and nutritious cereal-based foods.

## Figures and Tables

**Table 1:** Ratios of control wheat and intercropped flours in cake formulations.

Formulation	Control (WF)	IM	G72
Control (WF)	100		
IM (50%)	50	50	
G72 (50%)	50		50
IM (40%)	60	40	
G72 (40%)	60		40
IM (30%)	70	30	
G72 (30%)	70		30

Control (WF) = (control wheat flour), IM = non-germinated intercropped flour, G72 = 72 h germinated intercropped flour.

**Table 2:** Physicochemical, functional, pasting properties, and Texture Profile Analysis (TPA) of gels derived from intercropped mix flours.

Flour	Moisture (%)	Protein (%)	SDF (%)	WHC (g/g)	a*	b*	PV	FV	PT	Hardness (g)
**IM**	11.93 ± 0.03 ^f^	23.12 ± 0.46 ^a^	1.52 ± 0.90 ^a^	1.07 ± 0.00 ^c^	−8.37 ± 0.15 ^a^	21.53 ± 0.34 ^c^	806.00 ± 7.07 ^c^	1228.00 ± 2.82 ^b^	5.27 ± 0.00 ^abc^	428.41 ± 12.12 ^b^
**S8**	8.80 ± 0.07 ^e^	23.65 ± 0.05 ^ab^	1.76 ± 0.20 ^ab^	0.99 ± 0.01 ^ab^	−7.88 ± 0.09 ^ab^	18.68 ± 0.26 ^b^	761.50 ± 7.77 ^b^	1267.50 ± 12.02 ^b^	5.47 ± 0.00 ^c^	457.88 ± 5.91 ^c^
**S16**	8.54 ± 0.04 ^d^	23.88 ± 0.35 ^abc^	1.81 ± 0.38 ^ab^	0.99 ± 0.04 ^ab^	−7.50 ± 0.59 ^bc^	18.13 ± 1.48 ^ab^	856.00 ± 18.38 ^d^	1361.00 ± 9.89 ^c^	5.30 ± 0.04 ^bc^	461.20 ± 0.50 ^c^
**G24**	7.16 ± 0.18 ^b^	24.38 ± 0.18 ^bc^	2.20 ± 0.06 ^bc^	0.97 ± 0.03 ^a^	−7.34 ± 0.60 ^cd^	16.78 ± 1.52 ^a^	987.50 ± 4.94 ^e^	1506.00 ± 1.41 ^d^	5.03 ± 0.04 ^a^	554.64 ± 3.61 ^e^
**G48**	7.81 ± 0.04 ^c^	23.95 ± 0.32 ^abc^	2.53 ± 0.015 ^c^	1.03 ± 0.03 ^bc^	−6.95 ± 0.20 ^de^	16.76 ± 0.45 ^a^	859.00 ± 4.24 ^d^	1331.50 ± 7.77 ^c^	5.10 ± 0.14 ^ab^	489.88 ± 5.60 ^d^
**G72**	6.23 ± 0.02 ^a^	24.96 ± 1.02 ^c^	2.67 ± 0.12 ^c^	1.06 ± 0.00 ^c^	−6.49 ± 0.18 ^e^	16.64 ± 0.55 ^a^	692.00 ± 35.35 ^a^	1088.00 ± 39.59 ^a^	5.20 ± 0.18 ^ab^	399.61 ± 3.82 ^a^

Intercropped mix flours (IM = non-germinated control, S8 = soaked 8 h, S16 = soaked 16 h, G24 = germinated 24 h, G48 = germinated 48 h, G72 = germinated 72 h); PV = peak viscosity, FV = final viscosity, PT = peak time (min). Data are mean ± SD and are significantly different (*p* < 0.05) as per Fisher’s Least Significant Difference test (LSD). Different alphabets in the superscript of each column indicate the significant difference between each parameter value at different process conditions.

**Table 3:** Proximate composition of sponge cakes prepared from control wheat flour and non-germinated (IM) and germinated (G72) intercropped flours.

Category	Protein (%)	Ash (%)	Insoluble Dietary Fibre (%)	Soluble Dietary Fibre (%)	Total Dietary Fibre (%)
**Control**	9.52 ± 0.05 ^a^	1.34 ± 0.10 ^a^	1.52 ± 0.02 ^a^	0.47 ± 0.15 ^a^	2.50 ± 0.24 ^a^
**IM (30%)**	11.62 ± 0.11 ^b^	1.56 ± 0.05 ^b^	2.15 ± 0.13 ^ab^	0.88 ± 0.19 ^ab^	2.63 ± 0.29 ^a^
**IM (40%)**	12.15 ± 0.11 ^d^	1.69 ± 0.04 ^cd^	3.50 ± 0.19 ^c^	0.98 ± 0.27 ^ab^	4.39 ± 0.01 ^bc^
**IM (50%)**	12.43 ± 0.18 ^e^	1.73 ± 0.00 ^d^	6.04 ± 0.12 ^e^	1.46 ± 0.61 ^bc^	7.50 ± 0.73 ^e^
**G72 (30%)**	11.91 ± 0.00 ^c^	1.59 ± 0.00 ^bc^	2.74 ± 0.09 ^b^	1.55 ± 0.00 ^bc^	3.65 ± 0.91 ^ab^
**G72 (40%)**	12.35 ± 0.01 ^de^	1.77 ± 0.05 ^d^	4.18 ± 0.81 ^d^	1.71 ± 0.24 ^c^	5.89 ± 1.06 ^cd^
**G72 (50%)**	12.80 ± 0.06 ^f^	1.79 ± 0.00 ^d^	4.76 ± 0.26 ^cd^	1.99 ± 0.16 ^c^	6.76 ± 0.43 ^de^

IM = non-germinated intercropped flour, G72 = intercropped flour germinated for 72 h. Data are mean ± SD and are significantly different (*p* < 0.05) as per Fisher’s Least Significant Difference test (LSD). Different letters in the superscript of each column indicate the significant difference between each parameter value at different process conditions.

**Table 4:** Specific volume, colour and C-Cell parameters of sponge cakes prepared from control wheat flour and germinated and non-germinated intercropped flours.

Category	Specific Volume (mL/g)	Delta E (ΔE) Crust	Delta E (ΔE) Crumb	Slice Brightness	Area of Cells (%)
**Control**	1.84 ± 0.03 ^ab^	1.95 ± 0.27 ^a^	0.34 ± 0.03 ^a^	112.50 ± 3.2 ^e^	52.83 ± 0.50 ^b^
**IM (30%)**	2.09 ± 0.06 ^d^	3.84 ± 0.60 ^b^	5.32 ± 0.59 ^d^	97.06 ± 1.43 ^c^	50.43 ± 0.20 ^b^
**IM (40%)**	1.90 ± 0.02 ^bc^	6.12 ± 0.39 ^c^	6.85 ± 0.60 ^e^	93.20 ± 0.96 ^b^	50.93 ± 1.06 ^b^
**IM (50%)**	1.85 ± 0.03 ^abc^	8.20 ± 0.40 ^d^	7.82 ± 0.57 ^f^	87.86 ± 2.74 ^a^	49.53 ± 0.23 ^a^
**G72 (30%)**	1.93 ± 0.08 ^c^	3.51 ± 1.20 ^b^	3.34 ± 0.26 ^b^	101.66 ± 1.40 ^d^	50.43 ± 1.25 ^b^
**G72 (40%)**	1.86 ± 0.02 ^abc^	6.53 ± 0.56 ^c^	4.21 ± 0.37 ^c^	94.23 ± 0.76 ^bc^	50.76 ± 1.34 ^b^
**G72 (50%)**	1.82 ± 0.01 ^a^	10.10 ± 0.27 ^e^	6.21 ± 0.86 ^d^	91.60 ± 1.66 ^b^	49.73 ± 0.50 ^a^

IM = non-germinated intercropped flour, G72 = intercropped flour germinated for 72 h. Data are mean ± SD and are significantly different (*p* < 0.05) as per Fisher’s Least Significant Difference test (LSD). Different letters in the superscript of each column indicate the significant difference between each parameter value at different process conditions.

## Data Availability

The original contributions presented in this study are included in the article/Supplementary Material. Further inquiries can be directed to the corresponding authors.

## Supplementary Materials

The following supporting information can be downloaded at: https://www.mdpi.com/article/10.3390/foods15152713/s1, Figure S1: ANOVA of the effect of non-germinated and germinated intercropped flour (IM and G72) fortification on the rheological properties’ storage modulus, loss modulus and loss tangent (G′, G″ and Tan δ) of wheat-based cake batters.
