# Supplementary material for: Impact of Intercropped Legume Flours on the Nutritional and Textural Attributes of Wheat Cakes: A Sustainable Approach to Enhanced Nutrition
Source: Foods. 2026 Aug 1;15(15):2713. doi: 10.3390/foods15152713 (PMC13466192; doi:10.3390/foods15152713)
Supplement: Supplementary file 1 [file foods-15-02713-s001.zip › foods-4411205-supplementary.pdf]

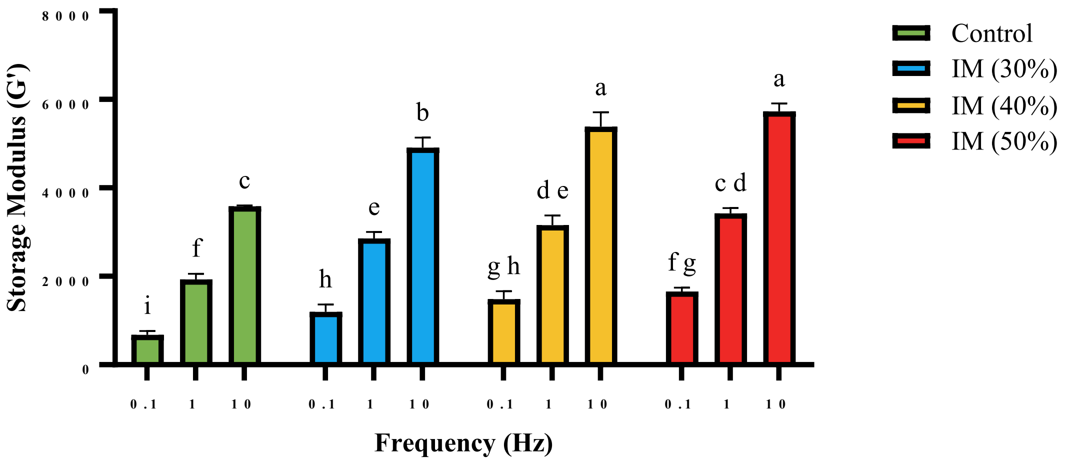
Storage Modulus (G')
8000
6000
4000
2000
0
i
f
c
h
e
b
g h
d e
a
f g
c d
a
0.1
1
10
Frequency (Hz)
Control
IM (30%)
IM (40%)
IM (50%)

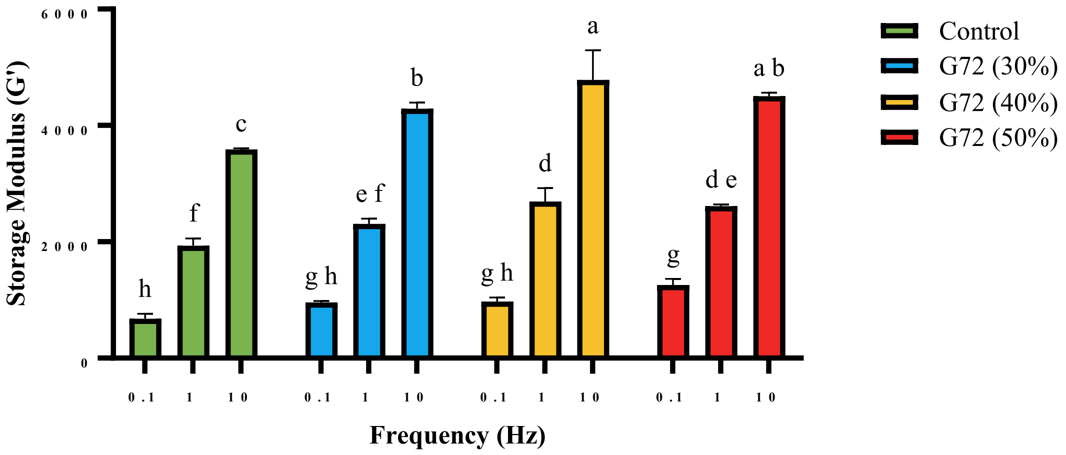
Storage Modulus (G')
6000
4000
2000
0
h
f
c
g h
e f
b
g h
d
a
g
d e
a b
0.1
1
10
Frequency (Hz)
Control
G72 (30%)
G72 (40%)
G72 (50%)

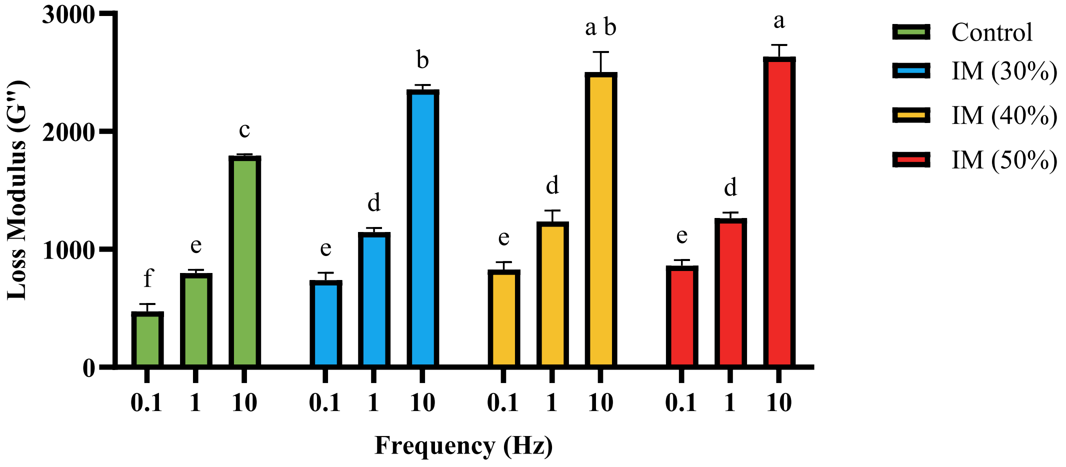

3000
2000
1000
0
Loss Modulus (G")
f
e
c
e
d
b
e
d
a b
e
d
a
0.1
1
10
0.1
1
10
0.1
1
10
0.1
1
10
Frequency (Hz)
Control
IM (30%)
IM (40%)
IM (50%)

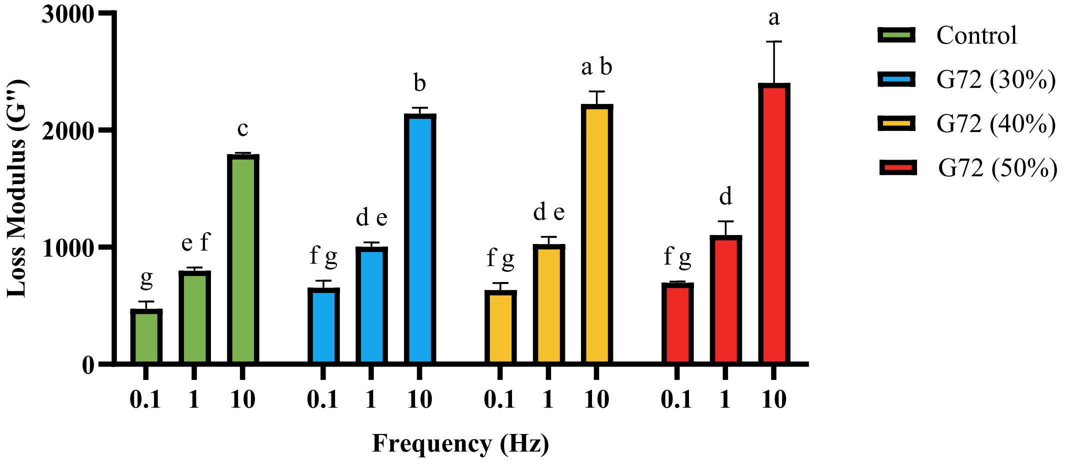

3000
2000
1000
0
Loss Modulus (G")
g
e f
c
f g
d e
b
f g
d e
a b
f g
d
a
0.1
1
10
0.1
1
10
0.1
1
10
0.1
1
10
Frequency (Hz)
Control
G72 (30%)
G72 (40%)
G72 (50%)

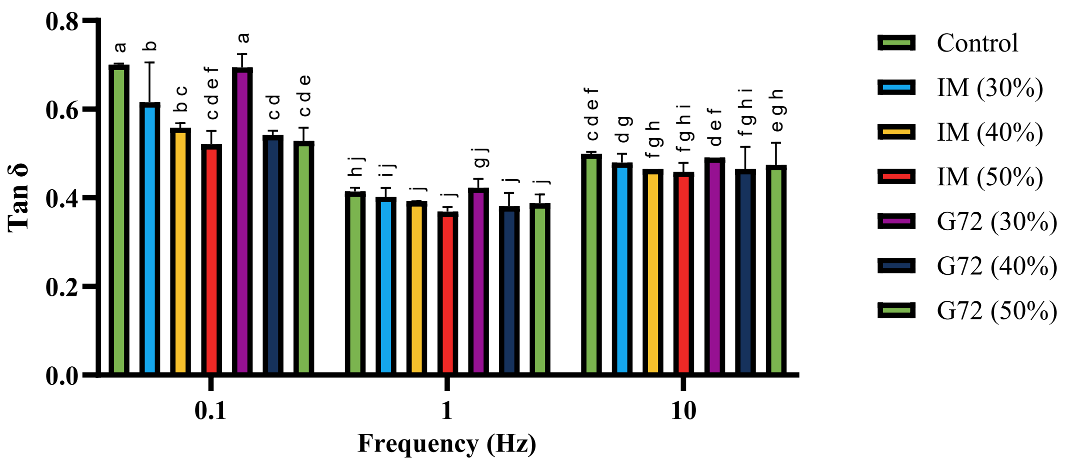

Figure S1: ANOVA of the effect of non-germinated and germinated intercropped flour (IM and G72) fortification on the rheological properties' storage modulus, loss modulus and loss tangent (G′, G″ and Tan δ) of wheat-based cake batters.
